# Cytogenetical studies in five Atlantic Anguilliformes fishes

**DOI:** 10.1590/S1415-47572009005000015

**Published:** 2009-01-16

**Authors:** Antonio Jales Moraes Vasconcelos, Wagner Franco Molina

**Affiliations:** Departamento de Biologia Celular e Genética, Centro de Biociências, Universidade Federal do Rio Grande do Norte, Natal, RNBrazil

**Keywords:** Anguilliformes, fish cytogenetics, Muraenidae, Ophichthidae

## Abstract

The order Anguilliformes comprises 15 families, 141 genera and 791 fish species. Eight families had at least one karyotyped species, with a prevalence of 2n = 38 chromosomes and high fundamental numbers (FN). The only exception to this pattern is the family Muraenidae, in which the eight species analyzed presented 2n = 42 chromosomes. Despite of the large number of Anguilliformes species, karyotypic reports are available for only a few representatives. In the present work, a species of Ophichthidae, *Myrichthys ocellatus* (2n = 38; 8m+14sm+10st+6a; FN = 70) and four species of Muraenidae, *Enchelycore nigricans* (2n = 42; 6m+8sm+12st+16a; FN = 68), *Gymnothorax miliaris* (2n = 42; 14m+18sm+10st; FN = 84), *G. vicinus* (2n = 42; 8m+6sm+28a; FN = 56) and *Muraena pavonina* (2n = 42; 6m+4sm+32a; FN = 52), collected along the Northeastern coast of Brazil and around the St Peter and St Paul Archipelago were analyzed. Typical large metacentric chromosomes were observed in all species. Conspicuous polymorphic heterochromatic regions were observed at the centromeres of most chromosomes and at single ribosomal sites. The data obtained for Ophichthidae corroborate the hypothesis of a karyotypic diversification mainly due to pericentric inversions and Robertsonian rearrangements, while the identification of constant chromosome numbers in Muraenidae (2n = 42) suggests a karyotype diversification through pericentric inversions and heterochromatin processes.

## Introduction

Cytogenetic analyses in fish have allowed to determine sex chromosomes (Moreira-Filho *et al.*, 1993; Devlin and Nagahama, 2002; Molina and Galetti, 2007), the characterization of vertebrate models, like the zebrafish (Sola and Gornung, 2001), the evaluation of genetically modified lineages (Porto-Foresti *et al.*, 2004), and to perform inferences on cytotaxonomic (Bertollo *et al.*, 2000; Bertollo *et al.*, 2004) and evolutionary issues (Demirok and Ünlü, 2001), besides the detection of cryptic species (Moreira-Filho and Bertollo, 1991). Nevertheless, cytogenetic data are still restricted for some fish groups, such as Anguilliformes, which comprises 15 families, 141 genera and 791 species (Nelson, 2006) and are popularly known as eels, congers or morays. Analyses of the 12S rRNA sequences support the monophyly of the Anguilliformes, but the phylogenetic relationships within the Order deduced from DNA analysis do not agree with those established through morphological comparisons (Wang *et al.*, 2003).

Previous chromosomal studies in Mediterranean moray species showed that constitutive heterochromatin was distributed on and around all the centromeres (Deiana *et al.*, 1990). A remarkable heteromorphism was reported between the NOR-bearing homologues in several species (Cau *et al.*, 1988), which in some cases led to the misidentification of this pair as sex chromosomes (Wiberg, 1983).

Although they represent some of the most typical reef fish groups in the Atlantic Ocean, few cytogenetic studies have been carried out in Muraenidae and Ophichthidae. In this work we performed a cytogenetic analysis of *Myrichthys ocellatus* (Ophichthidae), *Enchelycore nigricans*, *Gymnothorax vicinus*, *Gymnothorax miliaris* and *Muraena pavonina* (Muraenidae) collected in the Brazilian coast and around Atlantic oceanic islands, using conventional staining, Ag-NOR and C-banding.

**Figure 1:**
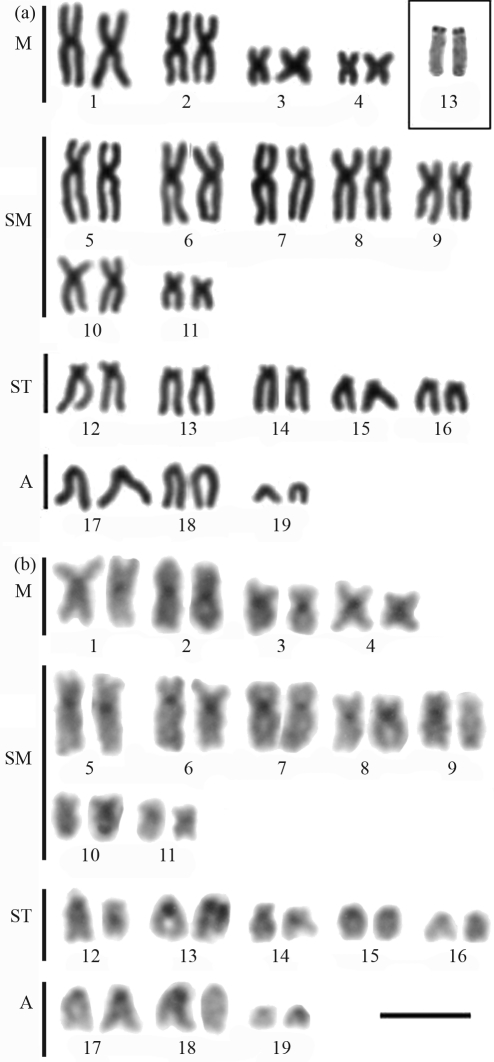
Karyotype of *Myrichthys ocellatus* after: (a) Giemsa conventional staining, Inbox, the NOR-bearing pair; (b) C-banding. Bar = 10 μm.

**Figure 2:**
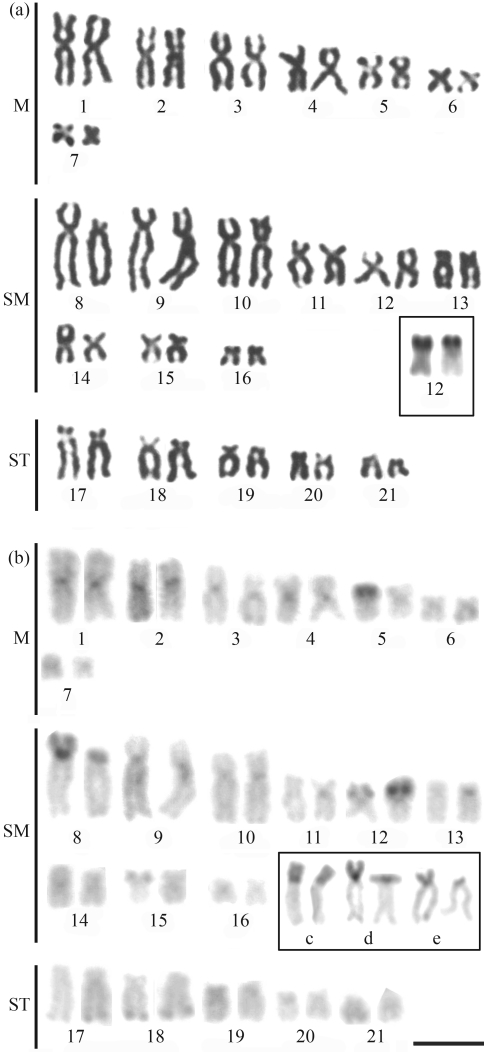
Karyotype of *Gymnothorax miliaris* after: (a) Giemsa conventional staining, Inbox, the NOR-bearing pair; (b) C-banding, Non-polymorphic (c-d) and polymorphic (e) heterochromatic regions on the short arms of pair 8. Bar = 10 μm.

**Figure 3:**
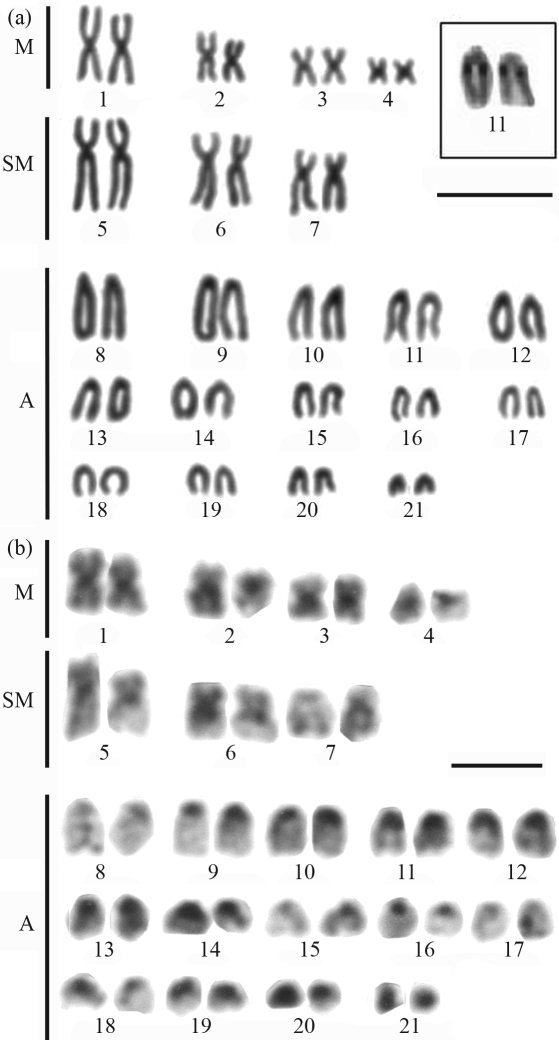
Karyotype of *Gymnothorax vicinus* after: (a) Giemsa conventional staining, Inbox, the NOR-bearing pair; (b) C-banding. Bar = 10 μm.

**Figure 4:**
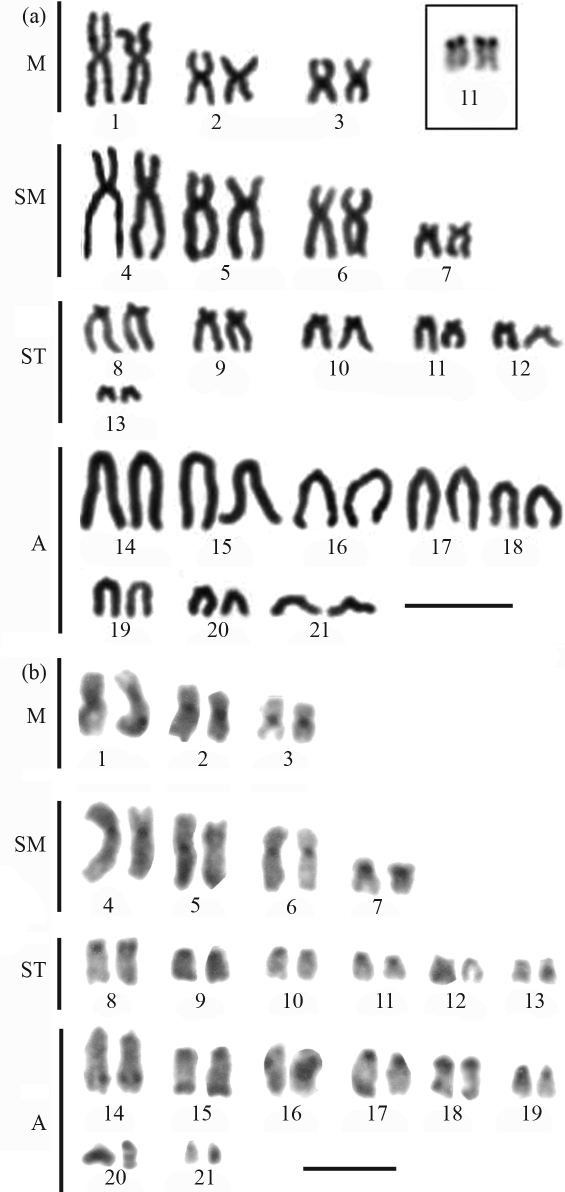
Karyotype of *Enchelycore nigricans* after: (a) Giemsa conventional staining, Inbox, the NOR-bearing pair; (b) C-banding. Bar = 10 μm.

**Figure 5:**
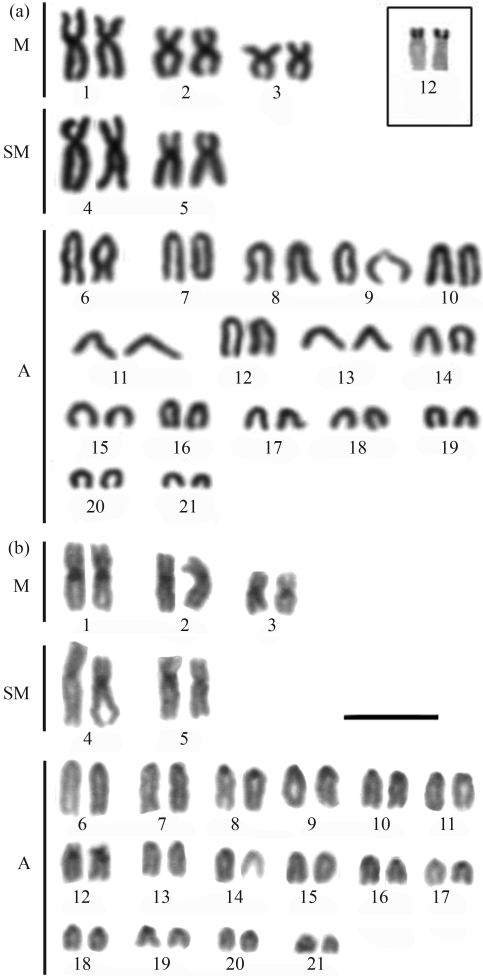
Karyotype of *Muraena pavonina* after: (a) Giemsa conventional staining, Inbox, the NOR-bearing pair; (b) C-banding. Bar = 10 μm.

## Material and Methods

The goldspotted snake eel *Myrichthys ocellatus* (12 specimens, undefined sex) and the purplemouth moray *Gymnothorax vicinus* (8 specimens, undefined sex) were collected along the shore of the state of Rio Grande do Norte (6°2'10" S/35°6'42" W), whereas specimens of golden-tail moray *Gymnothorax miliaris* (2 females) were collected in the coastline of Salvador (12°58' S/38°31' W), state of Bahia, northeastern Brazil. The viper moray *Enchelycore nigricans* (4 females and 2 males) and the white-spotted moray *Muraena pavonina* (6 females) were collected around the St Peter and St Paul Archipelago (0°55'02" N/29°20'42" W).

The individuals were mitotically stimulated for 24 h by intraperitoneal inoculation of either Munolan® (Allergan Frumtost), an association of fungal and bacterial antigens (Molina, 2002), or yeast suspension (*Sacharomyces cerevisae*) (Lee and Elder, 1980).

Chromosome preparations were obtained from kidney cells according to Gold *et al.* (1990). The sex of individuals was determined through macroscopic observation of gonads and histological analyses. The nucleolar organizer regions (NORs) were identified by silver nitrate staining (Howell and Black, 1980) and heterochromatic regions were evidenced after C-banding (Sumner, 1972).

The best metaphases were photographed using a digital system coupled to an Olympus BX42 microscope with 1,000X magnification. The chromosome pairs were arranged in decreasing size order and classified in relation to the centromere position as metacentric (m), submetacentric (sm), subtelocentric (st) or acrocentric (a) (Levan *et al.*, 1964).

## Results

The Anguilliformes species analyzed presented typical large chromosomes, ranging from 1.2 to 10 μm, and remarkably asymmetric karyotypes. Sex-related chromosomal heteromorphism was absent in *E. nigricans*.

*Myrichthys ocellatus*, the only Ophichthidae analyzed, presented a karyotype with 2n = 38 composed of 8m+14sm+10st+6a (FN = 70) (Figures 1a and b). A size heteromorphism unrelated to the NORs was sometimes present in the largest chromosome pair of this species (Figure 1a). Remarkable heteromorphisms were also present in the other analyzed species.

Amongst the Muraenidae, *Gymnothorax miliaris* showed 2n = 42 chromosomes, with 14m+18sm+10st and a high fundamental number (FN = 84) (Figures 2a and b). The homologues of pairs 1, 8 and 17 often presented significant differences in size (Figure 2a). A diploid number of 2n = 42 (FN = 56) and a karyotype formula with 8m+6sm+28a were observed in *G. vicinus* (Figures 3a and b). Some chromosome pairs, such as pairs 1 and 2, showed homologues of different sizes. The karyotype of *Enchelycore nigricans* was composed of 6m+8sm+12st+16a (FN = 68) (Figures 4a and b), while *Muraena pavonina*, presented a higher number of acrocentric chromosomes and a karyotype formula of 6m+4sm+32a (FN = 52) (Figures 5a and b).

All species presented single Ag-NOR sites, but located at different positions (see boxes in Figures 1 and through 5). Ag-NORs were located on the short arms of pair 13 (a) in *M. ocellatus*; in an interstitial position on the long arms of pair 11 (a) in *G. vicinus* and on the short arms of the pair 12 (sm) in *G. miliaris*. Ribosomal sites were identified on the short arms of pair 11 (st) in *E. nigricans* and on the short arms of pair 12 (sm) in *M. pavonina*.

C-banding revealed heterochromatic regions at the centromeric regions of all chromosomes in all the species (Figures 1 and through 5, b). After C-banding, a conspicuous size heteromorphism in the heterochromatin on short arms of pair 8 (Figure 2e) and on pair 12, coinciding with the Ag-NORs, was observed in one female *G*. *miliaris*. Telomeric heterochromatic segments were less frequent and were evidenced in *E. nigricans* (pairs 14 and 15) and in *G. miliaris* (pairs 12, 15, 17, 18 and 21) (Figure 2b).

## Discussion

A remarkable level of karyotypic diversification is found within Anguilliformes. The diploid number in this order ranges from 2n = 26 to 2n = 54 (Klinkhardt *et al.*, 1995), with variable karyotypic formulae and a high number of biarmed chromosomes.

Low diploid numbers (2n = 38) and a high number of meta-submetacentric chromosomes seem to be the most common condition for Ophichthidae species (Table 1). Species of the families Muraenesocidae, Congridae (Salvadori *et al.*, 1994), Anguillidae (Sola *et al.*, 1980; Sola *et al.*, 1984) and Echelidae (Amores *et al.*, 1995) also share a similar karyotypic pattern. Phylogenetic affinities based on the C- and G-banding patterns have also been proposed for the families Anguillidae and Congridae (Salvadori *et al.*, 1994).

A similar karyotype was also reported in *M. ocellatus* (2n = 38) that presented a typical karyotype formula, with mostly biarmed chromosomes (FN = 70). Discordant diploid numbers were identified in *Echelus uropterus* (2n = 50) (Nogusa, 1960) and *Muraenichthys gymnotus* (2n = 48) (Murofushi and Yosida, 1984). The karyotypical diversity reported in the family Ophichthidae has been mainly related to pericentric inversions and Robertsonian rearrangements (Takai and Ojima, 1985). A molecular phylogeny based on 12S ribosomal RNA sequences indicated Ophichthidae to be more derived than Muraenidae (Wang *et al.*, 2003).

Amongst Muraenidae, which comprises nearly 200 species, the available cytogenetic data suggest a basal diploid value of 2n = 42 with several acrocentric chromosomes (Table 1). This would be a basal condition when compared to other Anguilliformes (2n = 38). The variation in diploid numbers is smaller within this family, ranging from 2n = 36 to 2n = 42, with variable fundamental numbers (FN = 42 to 84). The karyotypes show a wide structural variation mainly due to pericentric inversions, which played a major role in the chromosome evolution of this species group.

*E. nigricans*, *G. vicinus* and *M. pavonina* present unique karyotypes that nevertheless show the pattern of the family Muraenidae with a high number of acrocentric chromosomes, a rare condition in other Anguilliformes. One exception was the karyotype of *G. miliaris* (2n = 42) that presented the highest FN reported so far in Muraenidae (FN = 84), likely due to pericentric inversions.

Karyotypes from both sexes were reported for only a few anguilliform species. Simple sex chromosome systems of the XX/XY type were reported in a muraenid, *Gymnothorax eurostus* (Takai and Ojima, 1985). A ZZ/ZW sex determination system was identified in some congrid species such as *Astroconger myriaster* (Park and Kang, 1979; Ojima and Ueda, 1982), *Conger japonicus* and *Alloconger anagoides* (Takai and Ojima, 1985) and in some species of the genus *Anguilla*, although some of these reports have been questioned (Wiberg, 1983; Sola *et al.*, 1984). Multiple sex chromosomes systems are rare within this group, but there is a description of a X1X2Y/X1X1X2X2 system in the ophichtid *Muraenichthys gymnotus*, where females presented 4st+44a (2n = 48) and males characterized by 1m+4st+42a (2n = 47) (Murofushi and Yosida, 1984).

According to Brum and Galetti (1997), diploid and fundamental numbers equal to 48 should be regarded as a synapomorphy for modern Teleosteans (Euteleostei) and Clupeomorpha. Since this trend is observed in these high taxonomic categories, the Anguilliformes (Elopomorpha) seem to have diverged from this pattern as a result of the reduction in the diploid number through chromosomal rearrangements, such as centric or in tandem fusions, followed by pericentric inversions.

In contrast with Perciformes, usually characterized by both numerical and structural karyotypic homogeneity (Molina, 2006), the Anguilliformes revealed structural chromosomal bands that suggest different levels of chromatin organization (Bernardi and Bernardi, 1990; Salvadori *et al.*, 1997; Pichiri *et al.*, 2000; Salvadori *et al.*, 2003).

Although the pattern of heterochromatin distribution is known for only a few Anguilliformes, the available reports indicate the presence of large heterochromatic blocks at pericentromeric positions or encompassing the whole length of the short arms of several chromosomes (Deiana *et al.*, 1990). Despite this, heterochromatic regions in *M. ocellatus* and *M. pavonina* were reduced and restricted to centromeric position on chromosomes. The Atlantic Muraenidae species *E. nigricans, G. miliaris* and *G. vicinus* presented a higher heterochromatin content with positive C-bands in nearly all chromosomes and some interspecific differences. These results are in agreement with previous studies carried out in this fish family (Cau *et al.*, 1988).

Although the compositional heterogeneity of heterochromatin has been commonly reported in fish (Souza *et al.*, 1996, among others), there are only few examples in marine species (Affonso and Galetti, 2005). Significant differences in heterochromatin composition have been reported in *Gymnothorax unicolor* and *Muraena helena* after chromosome digestion with restriction enzymes and CMA3 staining (Salvadori *et al.*, 1997), as well as through comparisons between MboI and 5S rDNA sequences (Pichiri *et al.*, 2000).

Heterochromatinization processes seem to have played an important role in the karyotypic evolution of Anguilliformes. Some studies have pointed out the relationship between a higher heterochromatin content and chromosomal diversity in fish species (Molina and Galetti, 2002; Molina, 2006). In *G. miliaris*, extensive heterochromatic polymorphisms could be observed in several chromosome pairs. Such polymorphisms involved an expansion of pericentromeric segments (homologues from pairs 2, 13 and 19), increase of the short arms (homologues of pairs 5, 8, 12 and 15) and differences in heterochromatin location between homologues (telomeric/centromeric, pairs 5, 11, 13 and 15).

In *G. vicinus*, size heteromorphisms were identified in pairs 1, 2 and 6, but could not be related to either differences in heterochromatin content or to NORs polymorphisms. Size heteromorphism and interindividual differences in centromere position were detected between homologues of some meta-submetacentric pairs of *G. miliaris*, *E. nigricans*, *M. pavonina* and *M. ocellatus*. The amplification of repetitive sequences led to changes in the morphology of many chromosome pairs. There are some reports of this type of heteromorphism within the order Anguilliformes, for instance in the species *Conger japonicus*, *Alloconger anagoides* and *G. eurostus* (Takai and Ojima, 1985).

Polymorphisms of the size of ribosomal sites have already been identified in Anguilliformes (Wiberg, 1983; Sola *et al.*, 1984). Ribosomal sites are present in a single chromosome pair (Salvadori *et al.*, 1994) and four patterns have been reported so far, all of them in Atlantic species: at a terminal location on the long arms of a submetacentric chromosome pair such as observed in *G. ocellatus* (Porto-Foresti *et al.*, 2005); at the telomeres of the short arms of a subtelo/submetacentric chromosome pair (as found herein in *M. ocellatus*; *G. miliaris* and *E. nigricans*); at a terminal position on the short arms of an acrocentric pair (such as in *M. pavonina*); and at an interstitial position, close to the centromeres on the long arms of an acrocentric pair (observed herein in *G. vicinus*).

The NOR pattern has been regarded as a potentially useful cytotaxonomic marker to species identification within Muraenidae (Salvadori *et al.*, 1994). Therefore, in *E. nigricans*, Ag-NORs were present on the short arms of a subtelocentric pair (11). In *G. miliaris*, ribosomal sites were identified on the short arms of a submetacentric pair at a telomeric position (12), and in *G. vicinus*, NORs were located interstitially on the long arms of an acrocentric pair (11), while in *M. pavonina*, Ag-NORs were observed on the short arms of an acrocentric pair (12). In the representative of the family Ophichthidae *M. ocellatus*, Ag-NORs were present on the short arms of pair 13 (acrocentric). Secondary constrictions equivalent to NORs were commonly observed in all analyzed species. Moreover, there was no association of heterochromatic segments with ribosomal sites, as previously observed in other fish groups (Artoni *et al.*, 1999).

Compared to Perciformes (Euteleostei), which often present a basal 2n = 48, many acrocentric chromosomes and a low heterochromatic content, the karyotypic pattern of Anguilliformes, as demonstrated in the present study, is characterized by large meta-submetacentric chromosomes and some large acrocentric elements, possibly originated through centric or in tandem fusions, as well as by heterochromatin accumulation. The karyotypic diversity among Atlantic morays and eels is reflected in their unique karyotypes, which can be used for cytotaxonomic purposes.

## Figures and Tables

**Table 1:** Cytogenetic data in species of Ophichthidae and Muraenidae (Anguilliformes).

Species	2n	FN	Chromosomal formula	Sex chromosomes	References
Ophichthidae					
*Echelus myrus*	38	58	20m-sm+18a	-	Salvadori *et al.*,1994; Amores *et al.*, 1995
*E. uropterus*	50	-	-	-	Nogusa, 1960
*Myrichthys ocellatus*	38	70	8m+14sm+10st+6a	-	*Present data
*Ophisurus macrorhynchos*	38	76	20m+14sm+4st	-	Nishikawa and Sakamoto, 1977
*Ophisurus macrorhynchos*	38	76	38m-sm	-	Vasil'ev, 1980
*Ophisurus serpens*	38	74	12m+24st+2a	-	Thode *et al.*,1985
*Pisodonophis boro*	40	40	40a	-	Natarajan and Subrahmanyam, 1974; Vasil'ev, 1980
*Pisodonophis boro*	38	64	18m+4sm+4st+12a	-	Khuda-Bukhsh and Barat, 1987
*Pisodonophis zophistius*	38	68	-	-	Nishikawa and Sakamoto, 1977
*Muraenichthys gymnotus*	48	52	4st+44a	X_1_X_1_X_2_X_2_	Murofushi and Yosida, 1984
*Muraenichthys gymnotus*	47	52	1m+4st+42a	X_1_X_2_Y	Murofushi and Yosida, 1984

Muraenidae					
*Enchelycore nigricans*	42	68	6m+8sm+12st+16a	No	*Present data
*E. pardalis*	42	52	8m+2sm+32a	-	Takai and Ojima, 1985
*Gymnothorax eurostus*	42	54	12m-sm+30a	XY	Manna, 1989
“	42	54	-	-	Takai and Ojima, 1986
*Gymnothorax miliaris*	42	84	14m+18sm+10st	-	*Present data
*Gymnothorax ocellatus*	42	76	16m+18sm+8a	-	Porto-Foresti *et al.*, 2005
*Gymnothorax reevesii*	42	76	-	-	Shoubai *et al.*, 1991
*Gymnothorax vicinus*	42	56	8m+6sm+28a	**-**	*Present data
*Gymnothorax kidako*	42	-	-	-	Nogusa, 1960
*Gymnothorax kidako*	42	-	-	-	Vasil'ev, 1980
*Gymnothorax kidako*	36	60	16m+8sm+12a	-	Taka and Ojima, 1986
*Gymnothoraxpictus*	42	42	42a	-	Rishi, 1973
*Gymnothoraxpictus*	42	-	-	-	Ojima, 1985
*Gymnothoraxunicolor*	42	54	12m-sm+30a	No	Deiana *et al.*, 1990
*Muraena helena*	42	60	18m-sm+24st-a	No	Cau *et al., * 1988
*Muraena pavonina*	42	52	6m+4sm+32a	**-**	*Present data
*Sideria picta*	42	42	42a	-	Takai and Ojima, 1985
